# Evaluating in the Real-World Educational Intervention to Improve Interference Control in Children with Autism Spectrum Disorder †

**DOI:** 10.3390/children9091294

**Published:** 2022-08-26

**Authors:** Elena Escolano-Pérez, Marian Acero-Ferrero

**Affiliations:** 1 Faculty of Education, Department of Psychology, University of Zaragoza, 50009 Zaragoza, Spain; 2 Department of Psychology and Sociology, University of Zaragoza, 50009 Zaragoza, Spain

**Keywords:** autism spectrum disorder, childhood, interference control, educational intervention, observational methodology, mixed methods, evaluation

## Abstract

Children with autism spectrum disorder (ASD) present deficiencies in interference control processes. The main aim of this pilot study was to analyze the efficacy of an educational intervention designed to optimize the interference control of eight ASD children, attending to their ASD severity level. A mixed-methods approach grounded in systematic observation and nomothetic/follow-up/multidimensional observational designs was used. An observation instrument was developed to code data, which were grouped according to the ASD severity level (Group 1, requires support; Group 2, requires substantial support) and were analyzed using a lag sequential analysis. The results show that, although both groups progressed during the intervention and could have continued to improve, each group evolved differently. Group 1 performed relatively well from the onset and increased and developed their interference control strategies throughout the intervention, while Group 2, despite also acquiring new interference control strategies, took more time to show improvements. One month after the intervention ended, both groups were unable to consolidate the strategies learned. A mixed-methods approach allowed for real interference control deficits in ASD children to be captured in a natural context. To conclude, it would be necessary to lengthen this intervention and adapt it to the needs of each group.

## 1. Introduction

Children with autism spectrum disorder (ASD) have persistent deficits in social communication and social interaction and display patterns of restricted and repetitive behavior, interests, and activities. Depending on the severity of the symptoms in these domains (and, therefore, how much support the person with ASD needs in his/her daily life), there are three levels of ASD (ASDL): ASDL1 = “requiring support”; ASDL2 = “requiring substantial support”; and ASDL3 = “requiring very substantial support” [1]. However, in addition to these core impairments, people with ASD present executive function deficits [2,3]. Executive functions are a set of general-purpose control processes that regulate one’s thoughts and behaviors. One of the fundamental components of executive functions is inhibitory control [4,5,6]. It refers to the process that enables controlling our actions, thoughts and attention, detaining automatic or dominant responses, and ignoring distracting stimuli in order to perform alternative behaviors according to the demands of the task or requirements of the context [4,5,6]. Inhibitory control is a multidimensional construct. It is composed of different subprocesses, separable but related. Several authors proposed diverse classifications regarding these inhibitory subprocesses [4,7,8,9,10,11,12,13], which can differ depending on the proposal adopted. Therefore, there is currently no consensus regarding which are the inhibition components or dimensions. However, some authors consider that, despite the particular terminology used by each taxonomy, there are some similarities among them [14,15,16]. One of these commonalities is to suggest that response inhibition and interference control are two dissociable subprocesses of inhibition control [4,7,8,9,10,11,12,13,14,15,16]. Response inhibition is the ability to suppress a dominant, prepotent, or automatic motor response [9,10]. Interference control refers to the ability to control one’s attention to select the information or stimulus needed to perform a particular task while ignoring distractors [10,17]. This last component, interference control, as compared to response inhibition, is somewhat less investigated in children with ASD [16], which justifies that this study is focused on it.

There is no consensus on the degree of involvement of interference control affectation in ASD. Some studies indicate that interference control is intact in ASD [2,18]. Others show that it is affected. Among them, again, there are discrepancies. Various works indicate that interference control is the only affected inhibition dimension [19]. Other research indicates that both dimensions (inhibition response and interference control) are affected, suggesting that there is a general impairment affecting all dimensions of inhibitory control in ASD, albeit in different ways [16,20]. These mixed results in the literature could probably be attributed to different sources of variability across studies related to: the characteristics of the sample (different intellectual functioning, comorbidity, age, gender); the assessment settings (laboratory or more natural settings, such as educational or clinical ones); the characteristics of the measures (computerized versus non-computerized or traditional tasks; direct versus indirect measures; motor versus verbal abilities required); and type of inhibitory control dimension assessed [16,21,22].

Executive functions, and, therefore, interference control, are malleable; that is, they can be improved through targeted training [4,6,23]. However, cognitive intervention programs aimed at improving these functions in ASD children are scarce, and there is no consensus regarding the results obtained [16,24,25,26,27]. In addition, there are still only a few programs whose effectiveness has been proven [26]. Furthermore, although follow-up assessments after the end of the intervention are critical to informing maintenance of training gains over time, and the literature recommends their inclusion, they are uncommon among the studies [26,27]. The scarce works containing follow-up assessments show a wide range of discrepant results [26,27,28]. Specifically, de Vries et al. [29] showed the training effects were maintained in some executive functions, such as working memory and cognitive flexibility, but not in inhibition control. Therefore, future research should include both immediate and follow-up measures to clarify this issue. Moreover, most of the studies that focus on executive function deficits in ASD individuals are developed in laboratory settings. To provide a more ecologically valid approach, there has been movement toward providing alternative assessments of ASD that are closer to everyday functioning in real-world settings [16,21]. This issue is very important, even more so when inhibitory deficits in ASD people could be more prominent in ecological contexts than in laboratory settings [16].

Systematic observation is the most pertinent and often the only methodology that allows for children’s skills to be captured in a natural context. Consequently, this methodology can be considered the most suitable for evaluation of interventions with young children in natural environments [30]. Systematic observation uses integrated quantitative and qualitative elements in (QUAL-QUAN-QUAL) macro stages, and, therefore, it is classified as a mixed-methods approach [30,31]. In an initial QUAL stage, an ad hoc observation instrument is created with consideration of the natural context, the objectives, and the observational design. The application of this observation instrument to the reality under study allows for the data to be coded on the basis of an order criterion. Precisely because it contains ordered information, the initial qualitative dataset can be transformed into quantitative data using different techniques—such as a lag sequential analysis—and, subsequently, a collection of quantitative results serving as sequential behavior patterns is achieved—(QUAN stage). Results are interpreted with consideration of the initial study problem (QUAL stage), which allows for seamless integration.

In the field of special education research, and particularly research relating to ASD children, the use of mixed methods has already been demonstrated [32].

With the purpose of contributing to the assessment of interference control in ASD children when they interact with adults, the aim of this pilot study was to analyze the efficacy of an educational intervention designed to optimize the interference control of eight ASD children, attending to their ASD severity level. To this end, the following was evaluated: (1) whether two groups of ASD children progressed during the intervention, and (2) whether the benefits remained one month after the intervention finished. Progress was considered based on whether one or various of the following characteristics were on the behavior sequential patterns obtained during the intervention: (a) an increase in correct inhibitory behaviors of children due to the adult help. These aids had to be progressively less directive as the intervention advanced or even disappear in favor of the appearance of various consecutive correct inhibitory child actions without adult intervention; (b) a new appearance or increase in changes of strategy to a correct inhibitory one; (c) the appearance of error self-detection; (d) the appearance of adjusted evaluations.

It was hypothesized that the intervention program would be effective. In other words, it was postulated that: (1) both groups of children with ASD would show progress during the intervention (i.e., their behavior patterns would be progressively characterized by the characteristics just mentioned); (2) these benefits would remain one month after the intervention finished (i.e., their behavioral patterns one month after finishing the intervention would retain these characteristics).

## 2. Materials and Methods

A mixed-methods approach—characterized by integrating qualitative and quantitative elements—was used [33]. More precisely, an observational methodology was used because it was the most suitable methodology considering the characteristics of our study and also because it is considered to be a mixed-methods approach itself [30,31]—as explained above. Considering the three forms in which integration data can occur as defined by Creswell and Plano Clark [34], the process of systematic observation is located in connecting, given that the sustaining procedure corresponds to the three major QUAL-QUAN-QUAL stages, with integration being located within the first two stages [35].

### 2.1. Design

In accordance with the classification of observational designs proposed by Anguera [30], the observational design used was nomothetic/follow-up/multidimensional. It was nomothetic because various units were studied; more specifically, 8 ASD children were observed individually. After, these observational data were analyzed jointly in two groups based on the severity level of each child. It was also considered a follow-up—at both the inter-sessional and intra-sessional levels—because 29 sessions were observed for each child and behavior patterns were recorded from the start to the end of each session. Finally, it was multidimensional since several levels of answer relating to the children’s interference control and the adult’s scaffolding behaviors were observed, which configured the observation instrument.

The systematic observation developed was guided by scientific criteria, non-participative (no interrelation between the observer and the observed participants), and direct (complete level of perceptibility of the events from the recorded video) [36]. This study follows the quality guidelines for studies based on observational methodologies [37], specifically the evaluative ones [38,39].

### 2.2. Participants

The participants were 8 Spanish male ASD children with ages ranging from 5 years 6 months to 12 years old (*M*_age_ = 92.38 months; *SD*_age_ = 21.74). Their IQ was between 52 and 110 (*M*_IQ_ = 81.63; *SD*_IQ_ = 21.98), their Working Memory was between 63 and 101 (*M*_WM_ = 81.87; *SD*_WM_ = 16.5), and their Processing Speed was between 71 and 91 (*M*_PS_ = 81.5; *SD*_PS_ = 9.5). Four of the participants showed ASDL1 characteristics (requiring support) based on the DSM-5 criteria [1]. Among them, 3 attended an ordinary school that provided preferential attention to students with ASD, and the other one received a combined education (i.e., he shared days between a school of the same type as the previous one and a special education school) [40]. The remaining 4 participants presented ASDL2 characteristics (requiring substantial support). All of them attended the same special education center (the same attended by the participant with combined education). Table 1 shows the specific characteristics of ASDL1 Group and ASDL2 Group. Based on parents’ information, no participant took any treatment, such as medication or any other evidence-based non-pharmacological intervention, or had behavioral problems.

Participants were selected from their schools based on the following inclusion criteria: (a) having a confirmed diagnosis of ASD by an expert clinician based on the DSM-5 (information provided by the parents); (b) having an additional confirmed diagnosis by a member of the research group using the Autism Diagnostic Observational Schedule-2 [41]; (c) being between 5 and 12 years old (information provided by the management teams of the schools and the parents); (d) having sufficient verbal abilities: score of 5 or less on each of the three dimensions—communicative functions disorder, expressive language disorder, and receptive language disorder—on the Communication and Language Scale from Autism Spectrum Inventory [42] (applied by the research group). The exclusion criteria were: (a) IQ ≤ 49 on the Wechsler Intelligence Scale for Children, Fourth Edition, in Spanish [43], according to the assessment realized by the research group; (b) having a diagnosis of physical disability and/or other mental psychopathologies (information provided by the parents).

This study was performed in accordance with the 1964 Helsinki Declaration and its later amendments or comparable ethical standards. The research was endorsed by the University review board and the management teams of the schools that the participants attended. All parents of the participants signed a written informed consent.

### 2.3. Program Intervention

The intervention to improve executive functions was designed considering the fundamental aspects indicated in the literature for effective training in this domain, especially with ASD children: (a) it must be close to real-world conditions, (b) it must be a play-based intervention, and (c) there must be an adult providing adequate support [23,44,45].

In accordance with the aim of this study, we focused on the tasks referring to interference control. These tasks were based on the Stroop paradigm, the most widely used one to evaluate interference control [46,47,48,49,50]. (However, it is also true that, because of the impurity problem of executive function tasks, there are some authors, such as Friedman and Miyake [11], who consider that the Stroop paradigm evaluates another inhibitory component: inhibition response.)

There are different Stroop tasks, but the standard version is the Stroop Color and Word. It was originally proposed by Stroop in 1935 [51] and involves the following. Firstly, color name words written in black ink are presented. The participant has to read the words. In the second phase, different colored rectangles are presented. The participant has to indicate the color of each stimulus. In the third and last phase, color name words are printed in different color ink from the word meaning (e.g., green printed in red ink). The participant has to indicate the color of the ink in which the word is printed (i.e., red in the previous example). Consequently, he/she has to resist or inhibit the distraction or interference generated by the written word and instead focus his/her attention on the color of the ink. Since this standard version of the Stroop test is not suitable for children (because it requires automatic reading), different versions of the Stroop task suitable for them have been created and frequently used in the literature: day-night task [52]; chimeric animal Stroop task [53]; color-object interference Stroop task [54]; pet store Stroop task [55]; or pictorial animal size Stroop task [56]. Based on these Stroop tasks for children, we created our intervention program tasks. There were 7 interference control tasks and one post-intervention task. Each one of the 7 interference control tasks included 8 activities with increasing difficulty. The difficulty level was determined according to the following issues [57,58,59,60,61,62]: (i) Number of stimuli and presentation time: Simple activities (S) = 10 stimuli were presented for 4 s; and Complex activities (C) = 20 stimuli were presented for 2 s [57,58]; (ii) Graduated indications provided by the adult to present the activity before the beginning of the child’s behavior: 1 = The adult offered modeling and asked the child to imitate her; 2 = The adult offered visual support; 3 = The adult offered auditive support; 4 = The adult did not provide any support [59,60,61]. Therefore, considering both criteria (i) Number of stimuli and presentation time with their two levels and (ii) Graduated indication provided by the adult with their four types-, the difficulty of the 8 activities ranged from less to more difficulty: S1, S2, S3, S4, C1, C2, C3, C4. The post-intervention task was composed of 3 activities shorter than those of the main intervention. Their difficulty level was C2, C3, C4. (Only complex activities were elaborated in the post intervention task because it is expected that, if a child is trained to master certain cognitive skills, those will allow him/her to develop higher level abilities [62].) This organizational structure of the tasks and activities can be consulted in Figure S1.

Regarding the content of the tasks and activities, it is necessary to clarify the following. Although all activities are aimed essentially to improve interference control, there are no pure activities in the intervention of executive functions that only train a single skill. Neither do real-life tasks usually involve a single or unique executive function but instead require an interplay of various executive functions [4,6]. Therefore, and according to literature that proposes that the activities used to measure inhibition involve different executive functions [63,64,65], the activities presented in the intervention require the simultaneous activation of other executive functions such as flexibility and working memory, preferred. The explanation of the content of the tasks and activities appears in Supplementary Materials (S1).

During the intervention, each child completed the 7 interference control tasks with their 8 activities. All the participants completed them in the same temporal order (which is indicated in Figure S1). Two activities were carried out in each session. The length of each session was around 30 min (ranging from 5 to 10 min depending on the abilities of the child), and its frequency was 2 sessions per week on non-consecutive days. Therefore, taking into account that it was 56 activities, the intervention for each child took place over 14 hour distributed in 28 sessions along 14 weeks (i.e., 3.5 months).

One month after the intervention finished, a post-intervention evaluation session was carried out. During this 30-minute session, the post-intervention task (with its corresponding 3 activities) was administered to each of the participants. The purpose of this post-intervention evaluation task was to consider the degree to which improvement in interference control had been maintained over time.

The participants received feedback of his execution in all the activities. These prompts were based on the self-instruction model [66] (see self-regulation prompts in Figure S2). Interventions based on self-regulation have demonstrated its effectiveness in children and adolescents [67]. Specifically, this model has been successfully implemented in children with autism [68] showing the opportunities of the learning in which errors are permitted and corrected with proper help, opposite the classic errorless learning [69]. Therefore, the monitoring process was included across the intervention, enhancing its intensity and generalization.

All sessions were carried out at the children’s schools by the same educational psychologist and were video recorded for subsequent viewing and analysis.

### 2.4. Instruments

The instruments used to identify the inclusion/exclusion criteria were the following: the Autism Diagnostic Observational Schedule-2 [41] to confirm an additional diagnosis of autism; the Communication and Language Scale from the Autism Spectrum Inventory [42] to confirm sufficient verbal skills; the Wechsler Intelligence Scale for Children, Fourth Edition, in Spanish [43] to discard an IQ ≤ 49.

To collect data, as systematic observation requires, an ad hoc observation instrument was created (available in the Table S1) and was entirely adjusted to the aims of the study. More specifically, this observational tool was developed to assess: (a) the executive functions of ASD children, and, more specifically, their interference control; and (b) the scaffolding behaviors shown by the educational specialist. This observation instrument is a combination of field format and categories system—based on the multidimensionality of the observational design used. This type of observation instrument allows for the recording of co-occurrent behaviors regarding multiple criteria [30].

To record the behaviors of the children and the adult in each session, a digital video camera was used. Behaviors were codified using the free software program LINCE [70]—available at http://observesport.com/ (accessed on 24 June 2022).

To analyze observational data quality (intra- and inter-observational reliability), SAS 9.1.3 [71,72] and GT software, v.2.0.E (Atlanta, GA, USA) [73] were used.

To carry out the lag sequential analysis, the software GSEQ5, v.5.1.23 (Arnstorf, Germany) [36] was used.

### 2.5. Procedure

The video recordings of the intervention and post-intervention sessions were transferred to the LINCE software and were coded using the observation instrument. The data were type II; that is, they were concurrent and event-based [74]—and multievent—because the study design was multidimensional, and the instrument of observation included field format and systems of categories [36].

The sessions were coded by two observers who were experts in executive functions and ASD. First, Observer 1 coded all the sessions, i.e., 232 sessions = (28 intervention sessions + the post-intervention session) × 8 children. Afterwards, 46 sessions were randomly selected to calculate reliability. This number of sessions was determined taking into account that the percentage of sessions mostly used by recent observational research to calculate the reliability ranges from 10 to 20% [75,76,77,78,79,80]. The sessions were chosen at random but taking into account that all the participants and all the tasks were reflected. Twenty-three of these sessions were coded by Observer 1 to calculate the intra-observer reliability. The other 23 sessions were coded by Observer 2 to calculate the inter-observer reliability. It involved that Observer 1 coded, in total, 127.5 recorded session hours and Observer 2 coded 11.5 h.

### 2.6. Data Quality Control Analysis

The intra- and inter-observer reliability were calculated through intraclass correlation coefficients (ICCs) using the generalizability theory [81]. SAS 9.1.3 and GT software were employed for the calculations. Regarding the intra-observer reliability, a three-faceted CA/M (Criterion, Category/Moment) design was used, and the analysis presented that the variability of the Moment facet was null (0%) in all sessions and that, in all cases, the ICCs were ≥ 0.96, indicating an excellent high intra-rater reliability. Regarding the inter-observer reliability, using a three-faceted CA/O (Criterion, Category/Observer) design, the analysis demonstrated that the Observer facet presented no variability at all and the ICCs were ≥ 0.92. Therefore, the inter-rater reliability was also high. Therefore, the interpretive rigor of the coding process was solvent.

### 2.7. Data Analysis

A lag sequential analysis [36,74] is a powerful statistical technique used to obtain associated relationships between codes based on a binomial test between conditioned and expected probabilities, applying the correction of Allison and Liker [82]. This test produces adjusted residuals that express the strength of the association between categories. Adjusted residuals are statistically significant for values > 1.96 for a significance level of *p* < 0.05 (indicating an excitatory or activating relationship between the categories). Therefore, starting from a concrete category (determined according to the aim of the study and the defined criterion or given behavior) allows us to identify which categories (conditional or target behaviors, which are also established in accordance with the aim of the study) occur in a backward direction (retrospective lag sequential analysis: lag −1, −2, etc.) and/or in a forward direction (prospective lag sequential analysis: lag +1, +2, etc.) with an occurrence probability greater than being random (adjusted residuals > 1.96 for *p* < 0.05; excitatory or activating relationship between the criterion and conditioned behavior) [83]. To interpret the patterns, Bakeman and Gottman [84] noted that (a) a pattern ends when two or more consecutive lags present non-significant behaviors; (b) a pattern weakens when two successive lags show multiple statistically significant behaviors (the first is the least interpretable one). This type of analysis has been successfully applied in interventions with ASD children [32,85] as it allows for change and continuity over the course of the intervention to be captured.

Regarding the aim of the study, participant records were combined based on their ASDL group (ASDL1 or ASDL2) and their corresponding task. In each group of participants, four tasks were analyzed: three intervention tasks and the post-intervention task. The three intervention tasks analyzed were upside-down game, crazy farm, and find my vehicle. The reason that justified the selection was the following. Analyzing these three intervention tasks allowed us to compare the interference control used by children at the beginning, middle, and end of the intervention since, considering the 14 weeks of the intervention, these tasks were administered on 1st and 2nd weeks (upside-down game), on 7th and 8th weeks (crazy farm), and on 13th and 14th weeks (find my vehicle). Therefore, analyzing these tasks permitted us to assess the improvement during intervention of each group. Analyzing the post-intervention task allowed us to determine the possible maintenance of the improvements made one month after the end of the intervention.

In order to carry out a lag sequential analysis for each ASDL group and task, according to the aims, we decided: (1) to select the following behavior criteria (marked in bold in the observation instrument—available in the Table S1); (1a) those children categories considered most related to interference control, appearing alone or co-occurring with others; and (1b) scaffolding strategies provided by the adult to support the children in carrying out the tasks; (2) to consider all the categories that configure the observation instrument as given behaviors (Table S1); (3) to apply a lag sequential analysis retrospectively (from −1 to −5 lags) and prospectively (from +1 to +5 lags). These chosen lags are the most habitually used [32,86] and allowed us to adequately reflect the complexity of our aims; (4) to set the level significance at *p* < 0.05.

## 3. Results

Table 2, Table 3, Table 4 and Table 5 show the sequential patterns of behavior of the ASDL1 Group, which were generated by both the children and the adult (as an executor) and obtained in upside-down game, crazy farm, and find my vehicle tasks, and in the post-intervention task, respectively, that is, the patterns that were generated at the beginning, middle, and end of the intervention, and one month after. Table 6, Table 7, Table 8 and Table 9 also represent this but in relation to the ASDL2 Group. In each table, the criteria behaviors appear in the central column. In the remaining cells, the conditional behaviors and the values of the statistically significant adjusted residuals are shown. Other relevant behaviors are highlighted: behaviors indicating progress in interference control are in green; wrong or inadequate interference control behaviors are in red; and adult behaviors are in blue.

In the patterns generated by the ASDL1 Group at the beginning of the intervention (Table 2), correct inhibitory behaviors (“CinUst”) generated patterns retrospectively and prospectively within more inhibitory behaviors (“CinUst”) and motivated help of the adult (Mh). These associations between behaviors (“CinUst” and “Mh”) also appeared when adult motivated help (“Mh”) was considered as a criterion behavior. Therefore, to execute and maintain correct inhibitory behaviors (“CinUst”), children had to be encouraged by the adult (motivating help of the adult, “Mh”). Some direct aids (such as “Rep” and “Ec”) were followed and preceded, respectively, with high probability, by infant error detection (“Ed”). Repetition (“Rep”) was also followed to correct inhibitory behaviors (“CinCst”), although with a lower probability. However, other helps, both direct and indirect (“Dh”, “In”, “Cm”), were not associated with interference control behavior (either correct or incorrect). In general, the patterns were dominated by adult behavior.

As the intervention progressed (Table 3), correct inhibitory behaviors that generated patterns increased: correct inhibitory strategies (“CinUst”) generated patterns (as at the beginning of the intervention) and also changes in behavior due to a correct inhibitory strategy (“CinCst”).

In this task, it was the first time that inhibitory errors were shown (“WinUst”, “WinCst”) to both generate patterns and be part of them. Some failures in activity tracking appeared in children who were not able to detect errors (“Ned”), sometimes occurring despite direct help (“Dh”) or indirect adult help (“Ih”). On some occasions (as at the beginning of the intervention), they detected the errors with adult help (“Ed”). The difference is that these aids were now less directive and included indirect aids (“Ih”) and reinforcement (“Mh”). Occasionally, the children detected errors by themselves (“Esd”).

At this point, some adult aids (“Dh”, “Ih”, and “Cm”), which, at the beginning of the intervention, were not associated with inhibitory responses (either correct or incorrect), were now associated with correct inhibitory responses (“CinUst”, “CinCst”). Therefore, these aids were more effective than at the beginning of the intervention. Reinforcement (“Mh”) continued (as at the beginning of the intervention) to be associated with correct inhibitory behaviors (“CinUst”) but now generated longer patterns. In addition, it was also associated with another type of correct inhibitory behaviors (“CinCst”). However, all the aids, except the directive aids (“Dh”), were also associated with inhibitory errors (“WinUst”, “WinCst”). Directive aids (“Dh”) were the only ones that were always associated with correct inhibitory behaviors (“CinUst”).

Finishing the intervention (Table 4), correct inhibitory behaviors (“CinUst” and “CinCst”) continued to generate patterns similar to those in the middle of the intervention. However, in this task, there were changes in two elements (inhibitory errors and quality of the children’s performance), which indicated substantial improvements in the children compared to the previous sessions.

Inhibitory errors were still present, but: (1) behaviors of change to a wrong inhibitory strategy (“WinCst”) no longer appeared. Thus, only wrong inhibitory strategies (“WinUst”) appeared as pattern generators or in a pattern. (2) These wrong inhibitory strategies (“WinUst”) were followed with high probability by error self-detections (“Esd”) or error corrections by changing to a correct inhibitory strategy (“CinCst”). They also appeared followed, although with lower probability, by other wrong inhibitory strategies (“WinUst”).

Regarding the quality of the children’s performance, there were important changes to be noted: (1) the predominance of the children’s behaviors in the patterns (and not the adult’s as at the beginning of the intervention) suggested an increase in the children’s autonomy. Adult reinforcement (“Mh”) and some punctual aids (“Ih”, “Ec”, “Rep”, “Cm”) were still present; however, the actions of the adult became a background activity. (2) Patterns showed that the autonomy of the children was linked to an improvement in their monitoring and evaluation skills. No error detection (“Ned”) and error detection with adult help (“Ed”) were not displayed in the patterns. Error self-detection (“Esd”) appeared in more patterns, and, moreover, appeared to generate patterns. Finishing the intervention for the first time, correct self-evaluation behaviors (“Aev”) appeared, although associated with adult help, as mentioned. All these aspects suggested an increase in complex cognitive skills in the ASDL1 Group.

As indicated, the effectiveness of indirect help (“Ih”) stood out. It was more effective for the children because it was followed with high probability by correct self-evaluation behavior (“Aev”). Other helps (“Mh” and “Cm”), associated with correct inhibitory strategies (“CinUst”, “CinCst”) at the beginning and/or in the middle of the intervention, were still linked. Moreover, “Mh”, in this final point of the intervention, was associated with error self-detection (“Esd”). Therefore, it was also more effective. Repetition (“Rep”), which was associated with correct inhibitory behaviors (“CinCst”) as at the beginning of the intervention, continued to be linked. However, it also appeared connected to wrong inhibitory strategies (“WinUst”). Certain direct aids (“Ec”, “Dh”) followed by correct inhibitory strategies (“CinCst”, “CinUst”) at the middle of the intervention were not yet effective.

One month after the end of the intervention (the post-intervention task; Table 5), both inhibitory behaviors (“CinUst”) and inhibitory behaviors with a change in strategy (“CinCst”) were maintained, and both generated patterns and were part of them. Patterns (as at finishing the intervention) showed the predominance of the children’s behavior, suggesting the maintenance of their autonomy. However, in this task, error self-detection (“Esd”) disappeared, reappearing again as error detection with adult help (“Ed”) and no error detection (“Ned”). In this regard, adult aids (“Dh”, “Ih”) were associated, with high probability, sometimes with children’s error detection (“Ed”), but, on other occasions, without detection of the error (“Ned”). Inhibitory errors (“Win”) did not appear. Despite these results, a slight regression appeared because the patterns were shorter and less relevant than during the intervention, which showed that the children’s behavior was not as sequential as in the intervention.

Regarding the sequential patterns of behavior of the ASDL2 Group generated both by the children and the adult (executor), the following aspects should be noted (Table 6, Table 7, Table 8 and Table 9).

At the beginning of the intervention (Table 6), the patterns, generated by both the children and the adult, showed that: (1) the ASDL2 Group performed very sporadic correct (“CinUst”) or wrong inhibitory behaviors (“WinUst”). (2) Furthermore, correct inhibitory behaviors (“CinUst”) only appeared to generate a pattern; in addition, this behavior appeared only associated with adult reinforcement (“Mh”), so the pattern obtained is very short. (3) The adult’s behaviors predominated (there were patterns formed by the adult’s behaviors only). (4) Different and frequent aids offered by the adult (“Ec”, “Dh”, “Ih”, “Cm”, and “Mh”) did not generate correct inhibitory behaviors in the children. (5) However, some aids (“Ec”) were effective for children to detect errors (“Ed”).

In the middle of the intervention (Table 7), correct (“CinUst”, “CinCst”) and wrong inhibitory (“WinUst”) behaviors were scarce. However, there were changes concerning correct inhibitory strategies (“CinUst”): they increased, generated more patterns (in which infant behaviors appeared associated), and were part of them (generated by both the children and the adult). Other improvements were: error self-detection (“Esd”) appeared and generated a change in strategy with correct inhibitory behaviors (“CinCst”)—which was also a new behavior that had not appeared previously. However, as at the beginning of the intervention, the different aids offered by the adult (“Mh”, “Cm”, “Dh”, “Rep”) did not enhance any inhibitory behaviors in children.

At the end of the intervention (Table 8), correct inhibitory behaviors (“CinUst”) increased. These inhibitory behaviors (“CinUst”) generated a significant and large pattern in which several behaviors of the same type appeared and were associated only with reinforcement (“Mh”). This pattern suggested important changes, not only because it generated a significant number of correct inhibitory behaviors but also because the children were able to benefit from this help (ineffective in previous tasks).

As at the beginning and in the middle of the intervention, wrong inhibitory behaviors were still present (“WinUst”). They increased their presence within a pattern. At the beginning of the intervention, errors were not always self-detected (“Esd”) as compared to at the middle of the intervention. At the end of the intervention, in some cases, children needed directive adult aids (“Ec”, “Dh”) to detect them. Therefore, these aids (ineffective in previous tasks) punctually increased their effectiveness because children in subsequent lags showed, with high probability, error detection (“Ed”) or error self-detection (“Esd”).

In summary, the most remarkable aspect of this task was that, as the intervention progressed, despite the errors, the children benefited from adult assistance and responded more effectively to the task.

One month after the intervention finished (post-intervention task; Table 9), wrong inhibitory behaviors (“WinUst”) disappeared. Correct inhibited behaviors (“CinUst”, “CinCst”) were still present. However, together with adult reinforcement (“Mh”), they were more punctual; that is, they did not enhance more behaviors of the same type. Therefore, children in this task did not benefit from these aids. Direct adult aids (“Dh”) were needed to enhance the change to a correct inhibitory behavior in the children (“CinCst”). Error self-detection disappeared (“Esd”). The children detected errors with help (“Ed”), and, sometimes, despite these aids, the children did not detect them (“Ned”). This is the first time this behavior appeared.

All these aspects indicate that the performances of the children, although they were not at the initial levels and they did not make mistakes, decayed substantially after one month without intervention.

## 4. Discussion and Conclusions

The observational methodology applied in this study—which is considered a mixed-methods approach—has contributed to a deeper understanding of interference control in ASD children and has done so by evaluating it in a natural context, which is an aspect that has been relatively unexplored so far [16]. It has brought us closer to a pending objective in ASD research: the development of an assessment instrument that enables collecting data on the functioning of children during an intervention without overloading the evaluation situation [87]. This observation instrument could be useful for professionals who work with ASD children. It can serve as a model or base that must be adapted or adjusted according to the characteristics of the natural context where each intervention takes place and the behaviors of interest, fulfilling the requirements of the observation methodology [30,37,39,88]. This adaptation will be necessary because observational methodology allows us to study behaviors that occur spontaneously or habitually in their own setting. Specific and diverse characteristics of each context will affect the habitual human behavior—the object of study—which will be very heterogeneous [88,89]. Providing this instrument is even more helpful if we take into consideration the literature that shows that many educational professionals still struggle to accurately assess ASD children’s performance [16].

In addition, the use of sequential analysis, which considers the flow of the action [36,74], has provided us information about the evolution of both groups during the intervention and not only before and after the intervention, as usually occurs when other methodologies and analysis techniques are used.

The patterns obtained in this research showed interesting results. Both groups of ASD children showed some improvements in some way during the intervention. However, even though both groups improved their interference control during the intervention, each one evolved in a different way and could have improved even further. Each group started from different points and reached different levels of execution. This is shown by the fact that, since the beginning of the intervention, both groups have shown correct inhibitory strategies (criterion behavior “CinUst”) with adult reinforcement (“Mh”). However, this criterion behavior in ASDL1 Group generated more correct inhibitory strategies (“CinUst”) from the beginning to the end of the intervention (see criterion behavior “CinsUst” in Table 2, Table 3 and Table 4). Instead, this achievement was not reached by ASDL2 Group until the end of the intervention (see criterion behavior “CinsUst” in Table 6, Table 7 and Table 8). Therefore, ASDL1 Group performed relatively well in the task at the beginning of the intervention, while ASDL2 Group took more time to show improvements. This shows different evolution trajectories between the groups.

Another issue to highlight is the role of the wrong inhibitory behavior in each group. At the beginning of the intervention, no inhibitory errors are shown in ASDL1 Group (see Table 2). This could be due to the difficulty level of the task (too easy to commit errors). In this regard, there are children with ASD that are able to overcome simple inhibitory tasks [12]. Therefore, it could be advisable to increase the difficulty level of this task for this group. In the middle of the intervention (see Table 3), inhibitory errors (“WinUst”) and inhibitory errors in changing strategy (“WinCst”) were both shown as a part of the pattern. Sometimes, children’s regulatory behaviors (“Esd”, “Ed”) or adult direct indications (“Dh”) made possible a change to another correct strategy (“CinCst”). At other times, the errors were not detected (“Nde”) and the errors remained (“WinUst”). At the end of the intervention (see Table 4), patterns with wrong behavior mostly contained self-regulation behaviors of the children (“Esd”) and even showed adjusted self-evaluation (“Aev”). Therefore, from the middle to the end of the intervention, error was a source of learning. Wrong behavior allowed to improve self-regulation strategies in ASDL1 Group. These results support the possibility of introducing errors with corresponding prompts as part of the intervention with children with ASD [69]. The benefits of self-regulated learning are also highlighted to achieve success in the task in children with ASD [68]. In ASDL2 Group, errors were shown since the beginning of the intervention (see Table 6), but, in this moment, they were not corrected, not even with adult direct help. At this point, it would be necessary to reduce the level of difficulty of the task. It seems to be higher than the children’s competential level. In the middle of the intervention (see Table 7), there was a changing trend since children’s correct behaviors were dominant and the adult behavior remained on a second plane. This indicates a significant improvement in autonomy in the children. Inhibitory errors generated a pattern in which children were able to detect their errors (“Esd”) (this behavior is presented with a high level of probability in the patterns) and also to change their behaviors for correct inhibited ones (“CinCst”). Finishing the intervention (see Table 8), there was some regression. The pattern generated by wrong behavior (“WinUst”) needed adult directive behavior (“Ec”, “Dh”) to obtain a correct answer. In this regard, the middle session could be analyzed in more detail to elaborate and to include in the intervention some similar activities to secure the acquired skills and to enhance autonomy and self-regulation in ASDL2 Group.

Regarding the effect of directive and non-directive aids of the adult on the children, the following was detected. There was great variability in the results throughout the intervention. At the beginning of the intervention in ASDL1 Group (see Table 2), some direct aids (such as “Rep” and “Ec”) were followed and preceded, respectively, with high probability by infant error detection (“Ed”). However, other helps, both direct and indirect (“Dh”, “In”, “Cm”), were not associated with interference control behavior (either correct or incorrect). In the middle of the intervention (see Table 3), some adult aids (both directive and non-directive, such as “Dh”, “Ih”, and “Cm”) are associated with inhibitory responses. Finishing the intervention (see Table 4), the effectiveness of indirect help was highlighted. Direct helps scarcely shows effectiveness. Differences observed in these patterns could also be due to differences in executive function requirements to succeed in the specific task/activity presented. An important source of variability could be due to the components each task demands. The modulating role of some components of the executive function, such as working memory or flexibility, may have modified the results [65,90]. In this line, the literature shows that resistance to distractor interference was closely related with other components of executive function, such as number generation performance, task switching ability, and everyday cognitive failures, all components that tend to be impaired in autism [90]. Another possibility would be that adult direct help did not adjust to the requirements of the children [91] because they were not effective at generating correct inhibitory behaviors. Therefore, finishing the intervention, it would be recommendable to readjust direct help. In ASDL2 Group, at the beginning and in the middle of the intervention, neither direct nor indirect help were effective to generate inhibitory correct responses (see Table 6 and Table 7). However, only while finishing the intervention was adult direct help effective to this group (see Table 8). Therefore, it seems recommendable to extend the intervention to this group. This could enhance the presence of more and better types of help to promote children’s autonomy.

Our results showed that, although both groups showed improvements after the intervention, ASDL2 Group is proportionally the one that obtained the most benefits. This group started from a lower execution level than ASDL1 Group, but they enhanced the inhibitory skills they had and acquired others that they initially lacked. They showed more changes throughout the intervention compared to ASDL1 Group. In this line, the intellectual functioning of each group could condition the implementation of the intervention. The intervention required different support for each group depending on their level of functioning [76]. These results are in line with the literature, which indicates that those with the poorest executive functions consistently gain the most from any program that improves these functions [92]. As was previously mentioned, ASDL1 Group’s behavior was already quite adequate at the beginning of the intervention, and they increased their inhibitory strategies and their autonomous use of these skills during the intervention; however, as with ASDL2 Group, they could have kept improving. The difficulty level of the tasks needs to be adapted throughout the intervention so that the children continuously train their skills at an optimal level [3]. As such, it would be necessary to include higher graduation of the activities. However, little is known about how to best manipulate task difficulty in interference control tasks [93], and more research is needed on this issue.

One month after the end of the intervention, the improvements achieved with the interventions were not fully consolidated in either group. In ASDL1 Group, some adequate and complex behavior patterns that had appeared at the end of the intervention no longer appeared in the post-intervention task. In ASDL2 Group, the adult aids remained the same, but the children reacted differently to them. From the beginning and throughout the intervention, ASDL2 Group had trouble generating inhibited behaviors with adult aids. In both groups, the patterns generated in the post-intervention task were shorter and no errors appeared. This highlights that it would be appropriate to increase the number of activities and the difficulty level for this task to aid the benefits of the children from learning with errors [69].

The literature found mixed results regarding effects of training on executive function in children in follow-up measures [27]. Our results are consistent with several studies evidencing that, once the intervention ends, the benefits diminish, although some effect remains [92,94]. This downward trend has been shown regardless of the type of intervention to improve executive functions [94]. The explanation for this situation is that, after stopping practice, due to the lack of stimulation, executive function will decline [94].

Therefore, an increase in executive functions certainly depends on the amount of time spent practicing. However, the optimal amount of practice to produce significant results has not yet been concluded [23]. Moreover, cognitive functions differ in terms of how easily they can be trained, and interference control may be one of the most difficult in this respect [92]. Consequently, interventions in interference control require more effort than interventions in other processes to produce appreciable and sustainable changes over time. As such, and in accordance with the results obtained, to be able to maintain the intervention effects, extending the intervention process timeframe for both groups is recommended, as well as re-graduating the levels of complexity for both groups, as already indicated.

Our study presents several strengths. (1) As previously mentioned, designing an interference control intervention and assessment that considers the natural school context of the children is an aspect to highlight in our study. Most studies that focus on the behavior of children with ASD are still being developed in laboratory settings even though it has not been widely proven that this is an accurate method for detecting real deficits in cognitive functioning and has not been shown to be an ecologically valid approach [16]. To truly achieve significant changes in the lives of ASD children, more observational studies should focus on their everyday functioning in the real world—as in this research. Moreover, developing school-based interventions is a way to provide accessible and low-cost treatment, especially to children from low socioeconomic situations who cannot access other interventions [95]. In this regard, designing school intervention implies contributing to the educational justice principle. Without intervention, there are numerous and relevant developmental consequences that can hinder possibilities. Equal chances to succeed in life for all children, of course, including those with disabilities and fewer economic opportunities, must be ensured [96]. (2) The confirmation of the initial formal diagnosis of the participants is another strong point of this study. It has involved overcoming a limitation indicated by the literature [13]. Some studies indicated that the ASD diagnostic criteria used by professionals can be partially changeable. As support for people with ASD (educational, medical) increases, the diagnosis or the severity level of the diagnosed deficits also increases. There are also apparent situations in the opposite direction. Specifically, in contexts where there is stigmatization of the disorder, the diagnosis or the severity level of the diagnosed deficits decreases [97]. Therefore, the use of ADOS—one of the gold standard assessments for ASD [98]—allowed us to ensure that the children belonged to each group or severity level. (3) This study introduced follow-up measures as an indicator of research quality [94]. The literature recommends this, but few studies have implemented it [26,27,99]. (4) The study considers the infant behavior in interaction with the adult observing behavior that helps or hinders success in the intervention [60,63,66].

This research has some weaknesses. (1) The first and major limitation is the reduced sample size. Research involving children is a lengthy and delicate process due to the inherent developmental characteristics of children and their specific vulnerability [100]; this is even more relevant when the children present special characteristics [101], just as ASD children do. These issues hinder access to these types of participants and justify the small sample size of our study. (2) All the participants were males. The fact that autism is four times more likely to occur in boys than in girls [96] could contribute to this aspect. Both mentioned issues (sample size and its composition) impeded the generalization of the obtained results to the whole population, even more so considering the known fact that, in the domain of executive functions and interference control, variability is very high, especially in ASD subjects [102]. (3) No instruments were used to confirm the information reported by parents, although research demonstrates the validity of information collected from ASD children’s parents [103]. (4) This pilot study analyzed the behavior of ASD children classified into two groups according to their severity level in order to examine the functioning of both groups. However, it would be beneficial to know the variability that characterizes ASD persons.

To overcome these limitations and support the reported results in this study, future studies should use larger and heterogeneous samples, including females, with further data. It would increase external validity. However, it should not be forgotten that, in observational studies, aiming to study a sample that is as large as those used in other methodologies is not possible due to the intensive (not extensive) character of observational methodology [88]. Referring to sex, some studies have reported sex-related differences in inhibitory performance [6], but other studies found no sex-related differences [12], and a sample with girls could be very relevant to clarify these inconclusive results. Considering the above-mentioned heterogeneity of ASD, it would be of interest to also analyze the children’s behavior individually. This would provide more information about the progress that each child achieves and, consequently, would allow for interventions to be more tailored to each child’s specific needs. In this regard, it would be necessary to consider the restricted interests of each child. The specific types of stimuli used in each task (animals, transports, etc.) could have a different impact on each child’s cognitive functioning depending on whether the stimuli are associated with their circumscribed interests [104]. Moreover, to analyze other different inhibitory components, such as inhibition response, and the other executive functions, such as working memory and shifting, in addition to other cognitive processes involved in the intervention tasks designed, is mandatory considering the complex interrelationship among all these processes [58,65]. It would offer more detailed information about ASD children’s cognition. To consider other factors that may be modulating infant interference control, such as IQ, age, or socioeconomic status [16,20], could constitute future research goals.

Unfortunately, many existing interventions regarding the executive functions of children with ASD have shown little to no evidence of generalized benefits, especially referring to interference control [58,99,105,106]. To understand how and under what conditions the medium- and long-term transfer of learning could occur is still a challenge, with important practical implications that necessitate further research [91,107].

In future studies, the complementary use of other analysis techniques, such as a “Markov analysis” [30,88], “polar coordinate analysis” [30,32,75,76,108], or a “temporal pattern (T-patterns) detection” [109], would offer greater information about interference control in ASD children.

Given the interference control deficits in autism spectrum disorder and the analysis of them in the real world—and not only in laboratory settings, which has been the most used context to date—early intervention may provide the best opportunity to alter the trajectory over an individual’s lifetime to improve the outcome for people with ASD. In this regard, it is a challenge to promote collaboration models between educational and research centers that promote practical research that is focused on both innovative pedagogical strategies and evidence-based interventions for the quality improvement of educational practice in the field of autism [98,110].

## Figures and Tables

**Table 1:** Participants’ characteristics: ASDL1 Group and ASDL2 Group.

	Age (Months)		IQ		WM		PS		ADOS		IDEA	
	M	SD	M	SD	M	SD	M	SD	M	SD	M	SD
ASDL1 Group	83	11	101.25	12.33	97	3.74	90.25	2.5	7.25	0.95	3.3	0.67
ASDL2 Group	101.25	29.78	62.00	10.46	66.75	3.5	72.75	1.7	10.5	1.29	4.3	0.53

WM = Working Memory; PS = Processing Speed; ADOS = Autism Diagnostic Observational Schedule-2; IDEA = Communication and Language Scale from Autism Spectrum Inventory; ADSL= Level of Autism Spectrum Disorder.

**Table 2:** Behavioral patterns obtained in the ASDL1 Group at the beginning of the intervention. Central column = It contains the criteria behaviors. Remaining cells = They contain the conditional behaviors. Numbers = Significant adjusted residuals values. Green codes = The most relevant behaviors indicating children progress in interference control. Red codes = The most relevant wrong interference control behaviors of the children. Blue codes = The most relevant adult scaffolding behaviors.

Executor	Lag −5	Lag −4	Lag −3	Lag −2	Lag −1	Criterion Behavior	Lag +1	Lag +2	Lag +3	Lag +4	Lag +5
ASDL1 Group	CDirCt 5.96	Ih 3.54	CDirCt 4.09	Rep 4.18	Ed 8.77	CDir	Ec 4.24	Ct 6.2	Ih 3.38	CDirCt 4.15	
				CinUst 2.21	Mh 2.16	CinUst	Mh 4.61	CinUst 2.98			
	Cm 8.49		UstWDir 8.43	Dh 8.6	Ih 3.18	UstCDir	Ec 6.12	CinCst 1.97			
				Cm 6.4		UstWDir	Dh 8.72	Ih 2.15	UstWDir 8.49	Ec 8.54	
Adult		UstWDir 5.92	Ih 3.26	Ed 4.18	UstCDir 6.16	Ec	Ct 8.72		CDirCt 5.96		
			Cm 5.92		UstWDir 8.77	Dh	Ih 2.59	UstCDir 8.83 Rep 2.15	Dh 5.96		
					UstWDir 2.71	Ih	UstWDir 3.83				
					CinUst 3.96	Mh	CinUst 2.02		CinUst 2.08		
						Cm		UstWDir 6.2	Dh 5.96		
	Ct 5.96		CDir 4.09	Ih 2.18	As 6.16	Rep	Ed 4.24	CDir 4.3	Ih 3.38	CinCst 1.99	

**Table 3:** Behavioral patterns obtained in the ASDL1 Group in the middle of the intervention. Central column = It contains the criteria behaviors. Remaining cells = They contain the conditional behaviors. Numbers = Significant adjusted residuals values. Green codes = The most relevant behaviors indicating children progress in interference control. Red codes = The most relevant wrong interference control behaviors of the children. Blue codes = The most relevant adult scaffolding behaviors.

Executor	Lag −5	Lag −4	Lag −3	Lag −2	Lag −1	Criterion Behavior	Lag +1	Lag +2	Lag +3	Lag +4	Lag +5
ASDL1 Group		Mh 2.34		Ed 2.25 CinUst 2.87	Mh 2.68	CinUst	Mh 6.14	CinUst 2.45	Ih 2.2		Ed 1.98
	Ih 3.49	Ed 2.3 Ned 5.86	WinUst 5.26	Esd 5.18	Ed 7.78 WinUst 3.35	CinCst		WinCst 5.16		UstWDir 5.12	Ed 2.74
	CstCDir 11.62		WinCst 4.08	CinCst 5.18		WinCst		Mh 1.98			
			Ned 3.58	Ih 2.98	Esd 3.6	WinUst	RNinCst 5.1	Ed 5.28 Ih 5.08			
					Mh 2.38	UstWDir		De 5.79			
Adult	Ed 2.29	CstDir 5.14	Ih 3.52	Per 5.18	CinCst 2.01 WinUst 7.2	Ec	WinCst 5.18	CinCst 1.99	WinUst 1.98		
				Ned 3.8	UstWDir 2.6	Dh	Ed 3.73 CinUst 3.22	CstCDir 2.58	CinUst 2.51	Mh 2.52	Esd 2.55
					Ned 4.69	Ih	Ned 4.67	WinUst 5.72		Ed 3.95	CinCst 3.48
	Mh 2.35	CinCst 2.15	CinUst 2.58		CinUst 6	Mh	UstWDir 1.99		Ed 1.97	CinUst 2.51	WinUst 2.87
	CinUst 2.36		CinCst 2.77		WinCst 6.76	Cm		CinUst 2.17	Mh 2.06		

**Table 4:** Behavioral patterns obtained in the ASDL1 Group at the end of the intervention. Central column = It contains the criteria behaviors. Remaining cells = They contain the conditional behaviors. Numbers = Significant adjusted residuals values. Green codes = The most relevant behaviors indicating children progress in interference control. Red codes = The most relevant wrong interference control behaviors of the children. Blue codes = The most relevant adult scaffolding behaviors.

Executor	Lag −5	Lag −4	Lag −3	Lag −2	Lag −1	Criterion Behavior	Lag +1	Lag +2	Lag +3	Lag +4	Lag +5
ASDL1 Group				CinUst 4.35	Ih 2.62 Mh 2.3	CinUst	Mh 4.17	CinUst 4.19		CinUst 2.18	
	Ct 6.1		WinUst 3.16	WinUst 7.43	Esd 8.74	CinCst	Mh 2.85				
	PerWDir 3.03	Ec 2.2	Ct 4.28	WinUst 3.16	Rep 4.1	WinUst	Esd 6.08	CinCst 5.6 WinUst 2.17	CinCst 3.55		
					WinUst 6.1	Esd	CinCst 11.31	Mh 3.25			
Adult	Ub 4.45	Ih 2.54	WinUst 2.55	Rep 7.89	WinUst 2.22	Ec	Ub 3.83				
				PerWDir 2.95	Ec 4.19	Dh	CDir 4.19				
					WinUst 2.01	Ih	Aev 4.08	CinCst 3.35	PerWDir 2.71	Esd 4.06	CinCst 3.83
	WDirCt 2.07	Cm 2.08	PerWDir 2.09	Esd 2.97	CinCst 2.42 CinUst 2.85	Mh	UstWDir 2.97				
	CinUst 2.09	Ih 2.54	CinUst 2	Mh 2.25		Cm	Mh 2.52	Ih 2.37	Ih 2.55	CDirCt 5.54	
	Ec 2.19	UstWDir 2.86	Ec 2.21	ErDirCt 4.29	WinUst 3.65	Rep	CinCst 2.22 WinUst 2.17	Ec 7.47			

**Table 5:** Behavioral patterns obtained in the ASDL1 Group one month after the intervention finished (post-intervention task). Central column = It contains the criteria behaviors. Remaining cells = They contain the conditional behaviors. Numbers = Significant adjusted residuals values. Green codes = The most relevant behaviors indicating children progress in interference control. Red codes = The most relevant wrong interference control behaviors of the children. Blue codes = The most relevant adult scaffolding behaviors.

Executor	Lag −5	Lag −4	Lag −3	Lag −2	Lag −1	Criterion Behavior	Lag +1	Lag +2	Lag +3	Lag +4	Lag +5
ASDL1 Group	UstCDir 2.83	UstCDir 2.66	UstCDir 2.55	UstCDir 2.34		CinUst	Mh 7.11				
	Ned 2.85	Ned 5.29 UstWDir 2.39	UstWDir 3.64	Ih 3.67	Ed 9.63	CinCst					
					UstCDir 3.73	UstCDir	UstCDir 3.61	CinUst 2.07 UstCDir 2.44	CinUst 2.13	CinUst 2.14	CinUst 2.16 Mh 2.47
						UstWDir	Ih 7.55	Ed 5.17 Ih 2.68	Ed 2.85	CinCst 3.59	
Adult					Ned 6.11	Dh	Ed 2.99 Ned 4.14				
					UstCDir 2.34	Ih	Ed 5.39 Ned 3.4	CinCst 2.86		Ned 3.55	
	UstCDir 3.24	UstWDir 2.39	CinUst 4.58	Mh 4.51		Mh					

**Table 6:** Behavioral patterns obtained in the ASDL2 Group at the beginning of the intervention. Central column = It contains the criteria behaviors. Remaining cells = They contain the conditional behaviors. Numbers = Significant adjusted residuals values. Green codes = The most relevant behaviors indicating children progress in interference control. Red codes = The most relevant wrong interference control behaviors of the children. Blue codes = The most relevant adult scaffolding behaviors.

Executor	Lag −5	Lag −4	Lag −3	Lag −2	Lag −1	Criterion Behavior	Lag +1	Lag +2	Lag +3	Lag +4	Lag +5
ASDL2 Group						CinUst	Mh 3.2				
						WinUst	Ih 3.48	WDir 3		Ec 2.18	Ih 2.71
Adult					Nae 4 WDir 4	Ec		Ed 2.64	CDir 2.64	Nae 3.91	Ec 2.71 Cm 2.61
		Dh 2.25		Dh 3.83		Dh		Dh 2.71			
					WinUst 3.67 Ek 2.71	Ih	Ec 2.5	Ec 2.97	Cm 2.67		CDir 3.88
	Ec 2.55	Ih 2.15		CDir 4.9	Mh 3.39	Cm		Ih 2.21	Ec 2.18		

**Table 7:** Behavioral patterns obtained in the ASDL2 Group in the middle of the intervention. Central column = It contains the criteria behaviors. Remaining cells = They contain the conditional behaviors. Numbers = Significant adjusted residuals values. Green codes = The most relevant behaviors indicating children progress in interference control. Red codes = The most relevant wrong interference control behaviors of the children. Blue codes = The most relevant adult scaffolding behaviors.

Executor	Lag −5	Lag −4	Lag −3	Lag −2	Lag −1	Criterion Behavior	Lag +1	Lag +2	Lag +3	Lag +4	Lag +5
				CinUst 2.11		CinUst	Mh 3.13				
ASDL2 Group			ApW 4.43	WinUst 7.87	Esd 7.94	CinCst					
					ApW 5.57	WinUst	Esd 7.87	CinCst 7.81			
Adult					CinUst 3.2	Mh	Dh 2.44		Dh 2.32		
						Cm	Rep 7.87	EkRc 5.48			

**Table 8:** Behavioral patterns obtained in the ASDL2 Group at the end of the intervention. Central column = It contains the criteria behaviors. Remaining cells = They contain the conditional behaviors. Numbers = Significant adjusted residuals values. Green codes = The most relevant behaviors indicating children progress in interference control. Red codes = The most relevant wrong interference control behaviors of the children. Blue codes = The most relevant adult scaffolding behaviors.

Executor	Lag −5	Lag −4	Lag −3	Lag −2	Lag −1	Criterion Behavior	Lag +1	Lag +2	Lag +3	Lag +4	Lag +5
ASDL2 Group		CinUst 2.91	Mh 2.16	CinUst 2.16	Mh 2.78	CinUst	Mh 3.25	CinUst 2.6		CinUst 2.24	
	WinUst 7.75	Dh 3.79		UstWDir 7.84	Esd 11.18	CinCst	Ct 5.5				
	Dh 2.96	WinUst 5.43	Esd 7.81 ApW 2.73		Dh 3.01	WinUst	Ec 7.84 Dh 3	Ed 7.81	UstWDir 3.74	WinUst 5.41	
						UstWDir	Dh 4.28 Esd 3.77				
Adult		ApW 4.86		Dh 3.82	WinUst 7.87	Ec	Ed 11.14		WinUst 11.05	Dh 3.77	
	WinUst 2.49	Ec 3.79	UstWDir 2.51		UstWDir 3.62 CstCDir 2.54	Dh	CstDir 5.43 WinUst 2.53	Dh 3.81	Esd 3.79	CinCst 3.77	WinUst 2.48
	Esd 2.96	Mh 2.95	Esd 2.21		CinUst 3	Mh	Ap 3.96	Mh 2.12	CinUst 2.29	Ih 3.27	Ct 3.18

**Table 9:** Behavioral patterns obtained in the ASDL2 Group one month after the intervention finished (post-intervention task). Central column = It contains the criteria behaviors. Remaining cells = They contain the conditional behaviors. Numbers = Significant adjusted residuals values. Green codes = The most relevant behaviors indicating children progress in interference control. Red codes = The most relevant wrong interference control behaviors of the children. Blue codes = The most relevant adult scaffolding behaviors.

Executor	Lag −5	Lag −4	Lag −3	Lag −2	Lag −1	Criterion Behavior	Lag +1	Lag +2	Lag +3	Lag +4	Lag +5
ASDL2 Group						CinUst	Mh 3.56	EkRc 3.35			
	Mh 3.32		UstWDir 6.93	Dh 9.9	Ed 9.95	CinCst					
						UstCDir	UstCDir 2.49	UstCDir 2.76	CinCst 6.89		
		Mh 2.35		Mh 4.79	Ned 7	UstWDir	Dh 6.96	Ed 6.93			
Adult	Mh 3.32		Mh 3.14		UstWDir 7	Dh	Ed 9.9	CinCst 9.85	Dh 3.13		
					CinUst 3.91	Mh	Ned 3.16	UstWDir 4.47		Ed 3.32 UstWDir 2.14	
			CinUst 4.04	Mh 3.76	ApW 5.59	Cm					

## Data Availability

The data presented in this study are available upon reasonable request from the corresponding author. The data are not publicly available due to privacy restrictions.

## Supplementary Materials

The following supporting information can be downloaded at: https://www.mdpi.com/article/10.3390/children9091294/s1, Figure S1: Organizational structure of the intervention. Figure S2: Self-regulation prompts. Table S1: Observation instrument. Supplementary File S1: Explanation of the tasks and activities of the program intervention.
