# Supplementary material for: Evaluating in the Real-World Educational Intervention to Improve Interference Control in Children with Autism Spectrum Disorder
Source: Children (Basel). 2022 Aug 26;9(9):1294. doi: 10.3390/children9091294 (PMC9497143; doi:10.3390/children9091294)
Supplement: Supplementary file 1 [file children-09-01294-s001.zip › Supplementary material-8.26/S1_Explanation of the tasks and activities of the intervention.pdf]

Explanation of the tasks and activities of the Program intervention

The intervention -composed of 7 tasks and one post-intervention task- is based on real life situations. The tasks are presented according to an increasing order of cognitive demands for the children.

As Figure S1 shows, each one of the 7 interference control tasks include 8 activities with increasing difficulty. The difficulty level was determined depending the following issues [53-55]: i) Number of stimuli and presentation time: Simple activities (S) = 10 stimuli were presented for 4 seconds; and Complex activities (C) = 20 stimuli were presented for 2 seconds; ii) Graduated indication provided by the adult to present the activity before the beginning of the child's behavior: 1 = The adult offered modeling and asked the child to imitate her; 2 = The adult offered visual support; 3 = The adult offered auditive support; 4 = The adult did not provide any support [55,56] . So, considering both criteria -i) Number of stimuli and presentation time -with two levels- and ii) Graduated indication provided by the adult -with four types-, the difficulty of the 8 activities ranged from minor to most difficult: S1, S2, S3, S4, C1, C2, C3, C4. The post-intervention task was composed of 3 activities shorter than those of the main intervention. Their difficulty level was C2, C3, C4. (In the post intervention task only were elaborated higher level activities because it is expected that, if a child is trained to master certain cognitive skills, those will allow him/her to develop higher level abilities [57]).

Although all activities are aimed essentially to improve interference control, there are no pure activities in the intervention of executive functions that only train a single skill. Neither do real-life tasks usually involve a single or unique executive function but instead require an interplay of various executive functions [4,6]. Therefore, and according to literature that proposes that the activities used to measure inhibition involve different executive functions [58-60], the activities that are presented in the intervention require the simultaneous activation of other executive functions such as flexibility and working memory, preferred.

The tasks are:

- **Task 1. Upside-down game**: It was elaborated following the day-night task [48].
    - Simple activities*:* it measures the inhibitory control capacity of an answer. Also, it requires the learning and memorization of two simple rules, its sustenance and control of interferences that avoids distraction. Two images are shown: one with a clean boy, and another one with a dirty boy. It is requested of the child to say the opposite to the image presented. When the clean boy is shown, he has to say “dirty”, and when the dirty boy is shown, he has to say “clean”. This version shows advantages that makes it more appropriate for the use in children: a) the stimuli are presented in the format of one page and not with cards; b) the stimulus is familiar to the boy; c) the children have to answer the stimulus with the correspondent name for the image (clean-dirty), decreasing the grade of required abstraction, which increments the automatic character of the answer.

- Complex activities*:* two images are shown to the child: one with a clean boy and another one with a dirty boy. The child is asked to say "handsome" when the dirty boy is shown and "ugly" when the clean boy is shown. This level requires more abstraction level since images -dirty boy and clean boy- do not represent completely the characteristic-ugly and handsome-

Executive demands: this task requires a certain demand of working memory to retain the opposite order and a certain flexibility level to adapt the changing demands of the stimulus.

- **Task 2. Slippers**: it is based on day and night task [48].
  - Simple activities: the child must name the color of the slippers he sees excepting when he sees a white one that he has to say "black".
  - Complex activities: child must name the color of the slippers he sees excepting when he sees a white one that he has to say black and when he sees a black one he has to say "white".

  Executive demands: compared to previous task, this task demands more charge in working memory, flexibility and interference since there is a higher number of stimuli to attend differently in each case, although not all require interference control.

- **Task 3. Big and small animals**: it is based on pictorial animal size Stroop task [34].
  - Simple activities: 4 animals are shown. Their size in images do not correspond to their real size (e.g. image of a big fly and a little elephant). When the child sees the image, he has to say "big" o "small" depending on the real size but ignoring the image he is seeing.
  - Complex activities: 8 animals are shown. Their size in images do not correspond to their real size. These 8 animals are with another with their real size.

  Executive demands: in this task there are several interferent stimuli and another non interference, so flexibility cognitive demands are increasing and also in working memory.

- **Task 4. The crazy farm:** it is based on the chimeric animal Stroop task [31].
  - Simple activities: black and white drawn animals are shown. Their heads do not correspond to their bodies (e.g. a sheep head with a cat body).
  - Complex activities: black and white drawn animal are shown. In this case the animal's head is painted in its real color (e.g. a yellow duck head) and the body of the animal is in black and white drawn. The child is asked to name the animal of the body it is shown.

  Executive demands: this task requires double inhibitory effort because the child is asked to identify the animal taking into account their body and ignore its head (heads facilitate the identification because the bodys are more similar). Secondly, in the complex activity the head is painted of its real color, this stimulus is more attractive (and this is the one that has to be inhibited) that the black and white drawn body (preferential stimulus).

- **Task 5. The lier**: it is based on the chimeric animal Stroop task [31].
    - Simple activities: a character appears with a squared head and body. Its head is colored one color and its body is also colored but with a different one. The child is asked to name the color of its bodies.
    - Complex activities: shape dimension is added to form dimension. So, these characters have a squared head and rounded body or rounded head and squared body with non-coincident colors in body and head. The child is asked to name the body shape of the characters.

  Executive demands: this task comparatively to previous one increment interference and working memory and flexibility demands because it is necessary to attend two dimensions: color and shape. This task requires the children to ignore distractions and focus on relevant information in each case.

- **Task 6. Pictograms**: it is based on color-object interference Stroop task [32].
    - Simple activities: it is shown pictograms with daily live objects colored of a different color as real (e.g. Grass red coloured). The child has to say the object and the color is colored (e.g. Red Grass).
    - Complex activities: it is shown a pictogram with daily live objects colored of a different color as real, but in this case the child is asked to name the object and its real color (e.g. a pictogram with a grass red coloured and he must to say green grass).

  Executive demands: in the simple activity though the child has to say what he sees, conflict consists of he must inhibit the impulse to say the real color of the object. In the complex activity the change of order requires flexibility to provide another response with the same stimuli used in the simple activity. Visual interference of the non-canonical colored object competes with verbal message children must say the real color of the object. This task also demands in both activities working memory to keep on line the order to each one. Especially in the complex activity to remember the real color of the object.

- **Task 7. Find my vehicle**: it is based on the Pet Store Stroop task [33].
    - Simple activities: in the center of a picture it is shown a person performing a profession. Close to him are two vehicles, one of his profession and another one of different profession. The child is informed that the professional in the picture is disguised and he is another person. The adult says who is actually the profesional. Child must link the professional with the corresponding professional vehicle the adult has said. (e.g. “I´m a fireman -picture of a policeman- now, find my vehicle!”. Child links the fireman “disguised as a policeman” with the fire engine).
    - Complex activities: the same picture is shown. In the center there is a person performing a profession, but in this case, there are four vehicles close to him, one of them corresponds to the professional and the rest corresponds to another profession. The child is informed that the professional in the picture is disguised and he is another person. The adult says who is actually the profesional. Child must link the

professional with the corresponding professional vehicle the adult has said. (e.g. “I´m a fireman -picture of a policeman- now, find my vehicle!”. Child links the fireman “disguised as a policeman” with the fire engine).

Executive demands: In this task the interference generated is verbal-visual. The child has to prefer verbal messages ignoring visual distractions. This is a challenge for children with ASD who benefit from visual input more than verbal.[54].

- **Post-intervention task. Colored fruits**: it is based on modified Stroop test [50] in order to avoid a reading task, in which the words were replaced by fruits. This task required the child to name 4 fruits (strawberry, banana, orange, pear) colored in a non-canonical color and also name its real color (e.g. it is shown a red banana and children have to say yellow)
  Executive demands: visual interference of the colored object in a non-canonical color competes with the verbal message the child must say corresponding to the real color of the object. This task requires working memory to keep the orden in line and to remember the real color of the object.

During the implementation of the intervention all the participants received feedback of his execution (see *self regulation prompts* in Supplementary material Figure 2). These prompts were based on the self instruction model [61]. They lead to success in task performance.
