# Supplementary material for: Evaluating in the Real-World Educational Intervention to Improve Interference Control in Children with Autism Spectrum Disorder
Source: Children (Basel). 2022 Aug 26;9(9):1294. doi: 10.3390/children9091294 (PMC9497143; doi:10.3390/children9091294)
Supplement: Supplementary file 1 [file children-09-01294-s001.zip › Supplementary material-8.26/Table S1_OI e.g..pdf]

Table S1. Observation Instrument.

Criterion	Category	Code	Description and example
Adult´s previous behavior	Activity proposal	Ap	The adult performs introductory actions aimed both at the presentation of the material necessary for the realization of the activity and the explanation of the activity (e.g. "This is the upside-down game. When you see an orange, you must say lemon and when you see a lemon you must say orange").
	Ensuring child knows the activity	Ek	The adult checks that, after having explained the activity to the child, he has understood it (e.g. "Now tell me what you have to do in this game").
Scaffolding	**Show**	**Sh**	The adult issues a verbal and/or gestural behavior that show to the child how he has the activity (e.g. "Do you see? The picture shows a lemon, then I must say orange").
	**Command**	**Cm**	The adult issues a verbal and/or gestural order that serves to regulate the beginning of the activity (e.g. "You can start now").
	**Repetition**	**Rep**	The adult repeats in whole or in part, the same intervention that she has previously performed. This repetition can be verbal and/or gestural and occurs immediately after her last intervention. It includes the presentation of visual stimuli (e.g. "Remember we are playing the upside-down game").
	**Direct help**	**Dh**	The adult conducts directive verbal and/or gestural behaviors in order to fix the child attention to something. This help constitutes a concrete guide that points and directs the child towards the adequate resolution of the activity. This category also includes the specific answer to a question that the child issues about the activity (e.g. "You must say lemon when you see an orange. ¿What you have to say now?". The adult shows to the child a picture with an orange).
	**Error correction**	**Ec**	The adult carries out verbal and/or gestural behaviors that correct the child´s wrong action and explicitly show the correct answer (e.g. "The correct answer is lemon. You must say lemon, repeat with me…lemon" The adult shows to the child a picture with an orange).
	**Indirect help**	**Ih**	The adult uses a verbal strategy that itself does not contain the answer to the problem that arises, but that tries to modulate a more adequate access to the activity and/or promote the reflection on the part of the child about the resolution (e.g. "Are you sure?").
	**Motivating help**	**Mh**	The adult, to generate a correct behavior in the child, him verbally or offers attractive material to him (e.g. "Well done!").
Child´s previous behaviour	Waiting	W	The child can wait while the adult explains the activity and until the order is given to start it. The child remains attentive to explanation and his action does not interfere with the adult´s behavior. Although the child can carry out verbalizations related to the activity he is going to face, he respects the turn of the speech (e.g. While the adult explains the activity, the child nods his head).
	Anticipation	An	The child tries to start the activity before the adult has explained it or before she gives the order to start it (e.g. The child gets the pictures and says "lemon, orange, orange…").
	Evasion	Ev	While the adult is explaining the activity, the child performs a voluntary or involuntary behavior that causes him to be distracted and separates his attention from the explanation. These behaviors may hinder the understanding of the activity to be performed (e.g. While the adult explains the activity, the child is looking to his hands and playing with them).
	Recall	Rc	The child shows that he knows the rules of the game that the adult is presenting. These behaviors occur while the adult is explaining the activity, without respecting the turn of speech, and before the child begins his execution (e.g. While the adult explains the activity, child says "Ok ok…I get it …in the upside-down game I have to say the opposite I see ").

Facing the task	**Use of strategy**	**Ust**	First verbal or/and gestural child´s intentional behavior to solve the activity. This category includes all child´s correct or wrong attempts to solve a problem. Subsequent attempts to solve the activity in a different way regardless of its effect on the action, adequate or not, will be considered in the category *Change of strategy* (e.g. The adult shows a picture with an orange and the child says "lemon").
	**Change of strategy**	**Cst**	Subsequent verbal and/or gestural child´s intentional behavior to solve the activity after he firstly has tried a different one. This category shows the amount of child´s different responses and reflects the reaction of the child after a wrong answer (e.g. The adult shows a picture with an orange and the child says orange and after says "No…lemon!").
	Persist	Per	The child stays in the same answer he has done previously. This behavior hinders to generate new answer and learn from error. This category includes those child responses that are equal to those given immediately before. These perseverative responses can occur both during the execution, at the end of it, during the review of the task requested by the adult, or in the self-evaluation of the execution (e.g. The adult shows the same picture for a second time with an orange and the child says "Orange" again).
	Ask	As	Throughout the activity, the child reveals through gestural or/and verbal behaviors that he does not know how to do something or that he has doubts, and implicitly or explicitly he requests the adult´s help. This category includes those gestures that indicate that the child seeks adult support such as: stand during execution and look at the adult for approval or assistance, look at the adult and shrug (e.g. The adult shows a picture with an orange. The child alternately looks at the stimulus and at the adult. Finally, he says: "I don't know what to do").
	No response	NR	The child does not perform any action (either verbal or gestural) when the stimulus is presented. That is, he does nothing (e.g. The adult shows a picture with an orange and the child remains silent).
	Unrelated behaviour	Ub	The child voluntarily or involuntarily, performs a verbal and/or a gestural behavior that is not related to the activity and that interferes with its optimal performance (e.g. While the child is doing the activity, he emits an echolalia).
Executio n	**Correct direct**	**CDir**	The child performs an appropriate verbal and/or gestural behavior. This category may involve both intermediate correct behavior during the activity and those that involve completing it correctly. Correct behaviors that require inhibition will be considered in the category Correct *inhibitory* (e.g. The child says red when he sees a red sleeper).
	**Wrong direct**	**WDir**	The child performs an inappropriate verbal and/or gestural behavior. This category may involve both intermediate incorrect behavior during the activity and those that involve completing it incorrectly. Inadequate behaviors that require inhibition will be considered in the category Wrong *inhibitory* (e.g. The child says red when he sees a green sleeper).
	**Correct inhibitory**	**Cin**	The child performs an adequate verbal and/or gestural behavior suppressing prepotent response and focusing on the relevant information. This behavior constitutes an adequate adjustment between the child`s execution and the demands of the activity (e.g. The child says lemon when he sees a picture with an orange).
	Wrong inhibitory	**Win**	The child performs an inadequate verbal and/or gestural behavior without suppressing prepotent response and not focusing on the relevant information. This behavior constitutes an inadequate adjustment between the child´s execution and the demands of the task (e.g. During incongruent activity the child says lemon when he sees a picture with a lemon).
Monitori ng	Checking	Ch	The child verifies his own performance throughout the activity. It includes looks comparing his execution with the proposed model (e.g. During the activity the child looks at the supporting material-pictograms- with information about the task).
	Error self detection	Esd	The child finds for himself the mistake he has made. Subsequently, the child may: a) be able to solve this error (for example, by performing a different behavior, but adequate to solve the activity-which would imply the use of the category *Change to a correct strategy*-)(e.g. The adult shows a picture with an orange and the child says orange and after says "No…lemon!").; b) try to solve the error but not achieve it (for

			example, by carrying out a different behavior but inappropriate to solve the activity- which would imply the use of the category *Change to a wrong strategy-)* (e.g. The adult shows a picture with an orange and the child says "orange" wait for a second and says "no…fruit!").
	Error detection with aid	Ed	The child locates the mistake he has made thanks to the fact that the adult has verbally and/or gesturally pointed out the existence of it. Subsequently, the child may: a) be able to solve this error (for example, by performing a different behavior, but adequate to solve the activity- which would imply the use of the category *Change to a correct strategy-*) (e.g. The adult shows a picture with an orange, the child says orange and the adult says "are you sure?" then the child says "No…lemon!"); b) try to solve the error but not achieve it (for example, by carrying out a different behavior but inappropriate to solve the activity – which would imply the use of the category *Change to a wrong strategy-*) (e.g. The adult shows a picture with an orange, the child says orange and the adult says "are you sure?" then the child says "No…fruit!"); c) do not even try to solve his error (for example, avoid continuing in the activity -which would imply the use of the category *Unrelated behavior-*) (e.g. The adult shows a picture with an orange, the child says "orange", the adult says "Are you sure?" then the child starts to sing").
	Comments during the task	Ct	Related comments of the child about his own execution during the activity (e.g. "Am I doing in the right way?").
	No error detection	Ned	The child is not able to locate the mistake he has made (e.g. The child makes a mistake, but he continues the activity despite the adult asking him to check it).
Evaluatio n	Adjusted evaluation	Aev	The child makes an appropriate judgment when comparing his execution/result with the demand of the activity. This category implies both the judgment issues during the activity and at the end of it (e.g. The child is committing errors during the activity and he says: "I´m doing this game so badly").
	Non adjusted evaluation	Nae	The child makes an inappropriate judgment when comparing his execution/result with the demand of the activity. This category implies both the judgment issues during the activity and at the end of it (e.g. The child is committing errors during the activity and he says: "I´m doing this game very well)..

Note: Bold= Categories (and their codes) considered in the sequential analysis as the criterion behaviors (see explanation in the Data analysis section); In red: example of the category.
