# Supplementary figures and images for: Evaluating in the Real-World Educational Intervention to Improve Interference Control in Children with Autism Spectrum Disorder
Source: Children (Basel). 2022 Aug 26;9(9):1294. doi: 10.3390/children9091294 (PMC9497143; doi:10.3390/children9091294)

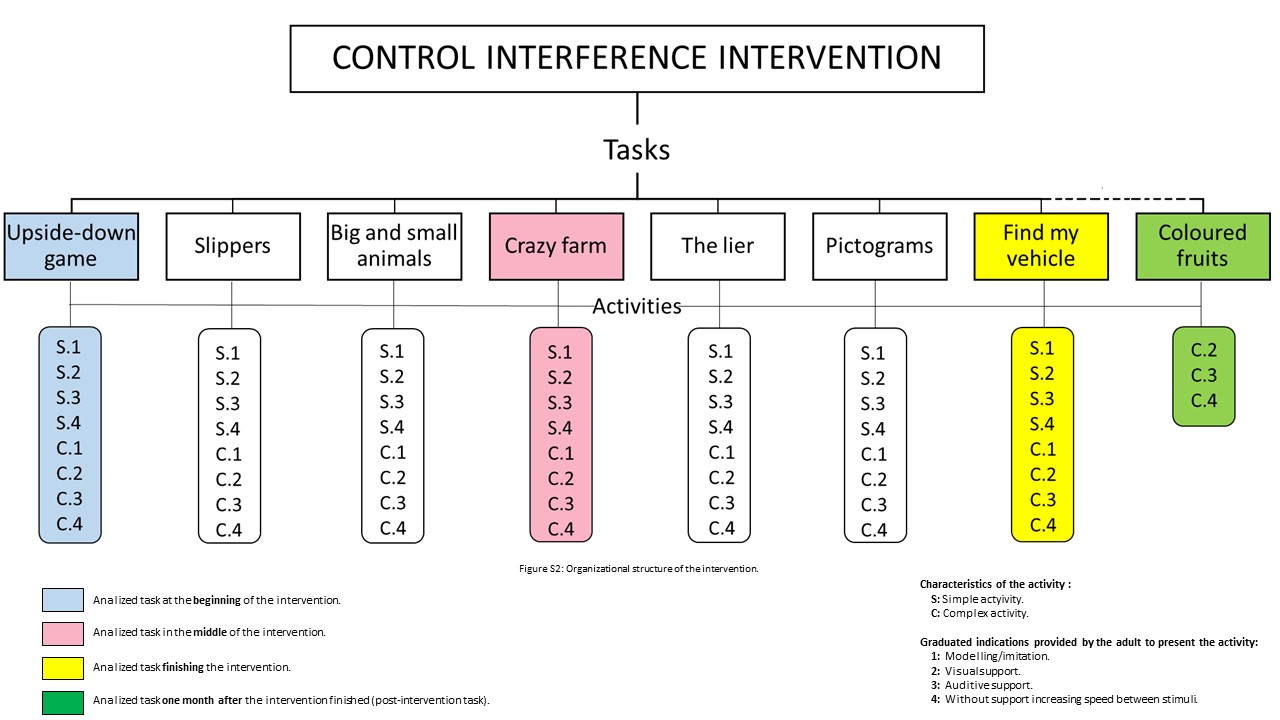

Supplement: Supplementary file 1 [file children-09-01294-s001.zip › Supplementary material-8.26/Figure S1_organizacional structure of the intervention.jpg]

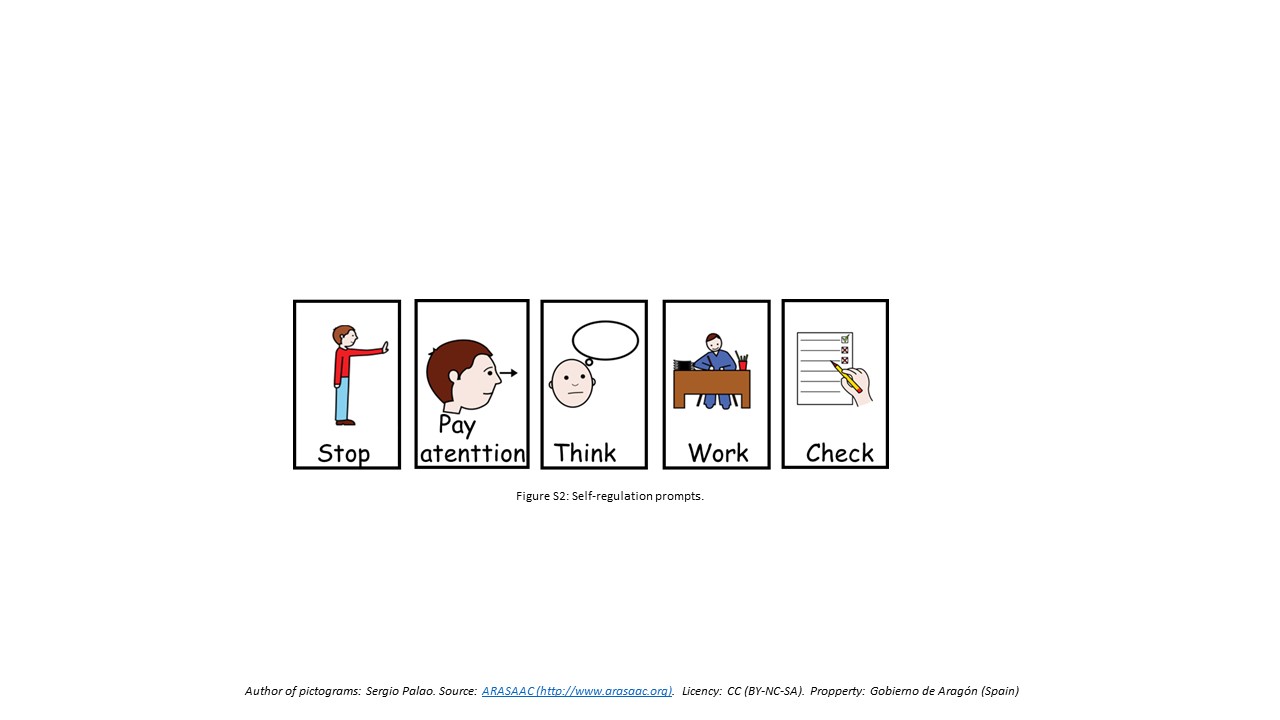

Supplement: Supplementary file 1 [file children-09-01294-s001.zip › Supplementary material-8.26/Figure S2_ self-regulation prompts.jpg]
